# Case Report: Left atrial myxoma with confirmed Delta variant COVID-19 infection, “to treat or withhold”

**DOI:** 10.12688/f1000research.124159.1

**Published:** 2022-08-31

**Authors:** Sally Aman Nasution, Eric Daniel Tenda, Eka Ginanjar, Nuri Dyah Indrasari, Ariel Pradipta, Mira Yulianti, Muhadi Muhadi, Anindya Pradipta Susanto, Muhammad Arza Putra, Moses Mazmur Asaf, Ceva Wicaksono Pitoyo, Imam Subekti, Ari Fahrial Syam, Lies Dina Liastuti

**Affiliations:** 1 Division of Cardiology, Department of Internal Medicine, Faculty of Medicine Universitas Indonesia, Dr. Cipto Mangunkusumo National General Hospital, Jakarta Pusat, DKI Jakarta, 10430, Indonesia; 2 Division of Respirology and Critical Illness, Department of Internal Medicine, Faculty of Medicine Universitas Indonesia, Dr. Cipto Mangunkusumo National General Hospital, Jakarta Pusat, DKI Jakarta, 10430, Indonesia; 3 Department of Clinical Pathology, Faculty of Medicine Universitas Indonesia, Dr. Cipto Mangunkusumo National General Hospital, Jakarta Pusat, DKI Jakarta, 10430, Indonesia; 4 Genomik Solidaritas Indonesia (GSI) Lab, Jakarta Selatan, DKI Jakarta, 12430, Indonesia; 5 Department of Surgery, Division of Cardiothoracic and Vascular Surgery, Faculty of Medicine Universitas Indonesia, Dr. Cipto Mangunkusumo National General Hospital, Jakarta Pusat, DKI Jakarta, 10430, Indonesia; 6 Division of Endocrine Metabolic and Diabetes, Department of Internal Medicine, Faculty of Medicine Universitas Indonesia, COVID-19 Board Dr. Cipto Mangunkusumo National General Hospital, Jakarta Pusat, DKI Jakarta, 10430, Indonesia; 7 Division of Gastroenterology, Department of Internal Medicine, Faculty of Medicine Universitas Indonesia, COVID-19 Board Dr. Cipto Mangunkusumo National General Hospital, Jakarta Pusat, DKI Jakarta, 10430, Indonesia; 8 Department of Cardiology and Vascular Medicine, Faculty of Medicine Universitas Indonesia, Dr. Cipto Mangunkusumo National General Hospital, Jakarta, DKI Jakarta, 10430, Indonesia

**Keywords:** COVID-19, delta variant, myxoma, primary cardiac tumors

## Abstract

Primary cardiac tumors (PCTs) are extremely rare entities. More than half of PCTs are benign, with myxoma being the most common tumor. Generally, simple tumor resection is the treatment of choice for benign PCTs since it has promising results that yield low complication and recurrence rates. However, in the COVID-19 pandemic era, the mitigation protocols and/or concurrent COVID-19 infection should be taken into account in patient management for the best overall outcome. To our knowledge, this is the first case report of a patient with a left atrial myxoma and systemic embolism complication in the form of an ischemic stroke, with a concurrent confirmed COVID-19 delta variant infection.

## List of abbreviations

CK: Creatine kinase

CK-MB: Creatine kinase – myocardial band

CPB: Cardiopulmonary bypass

CRP: C-reactive protein

FDG-PET/CT: 8F-fluorodeoxyglucose positron emission tomography/computed tomography

GCS: Glasgow coma scale

HCU: High care unit

ICCU: Intensive cardiac care unit

IVFD: Intravenous fluid drops

NSTEMI: Non ST-elevated myocardial infarction

PCT: Primary cardiac tumor

SC: Subcutaneous

TEE: Transoesophageal echocardiography

TTE: Transthoracic echocardiography

## Introduction

Primary cardiac tumors (PCTs) are an exceptionally rare abnormality. One study in the United States documenting autopsies found that the frequency of PCTs is around 0.02% (200 tumors in 1 million autopsies). ^1^ Another study found that PCTs only account for around 0.3 – 0.7% of all cardiac tumors. ^2^ Roughly 66.7% to 75% of all cardiac and pericardiac tumors are benign, while the rest are malignant. ^3^ Out of all the PCTs, atrial myxoma is found to be the most common benign tumor, ^4^^,^^5^ while angiosarcoma is the most common malignant tumor. ^3^^,^^5^ Approximately 70-75% of all cardiac tumors are myxomas, occurring predominantly in middle-aged females. ^5^^,^^6^ They usually arise out of the fossa ovalis, and the most common place for myxomas to appear is the left atrium (75%), followed by the right atrium (18-23%) and the ventricles (2%) respectively. ^5^^,^^6^ Although the clinical manifestations of cardiac myxomas are undefined since they are dependent on the size, location, and mobility of the tumor, patients with cardiac myxoma commonly have three main patterns of clinical presentation: haemodynamic consequences, systemic embolism, and constitutional manifestations. ^5^^,^^6^ Although the gold standard for the diagnosis of PCTs requires histological examination, multimodality cardiac imaging has shown promising results. ^6^^,^^7^ 18F-fluorodeoxyglucose positron emission tomography/computed tomography (FDG-PET/CT) was found to have a sensitivity of over 90% in differentiating benign and malignant processes. ^5^ Meanwhile, transthoracic echocardiography (TTE) and transoesophageal echocardiography (TEE) are suggested to have excellent sensitivity (95% and 100% sensitivity respectively) in detecting cardiac myxomas and differentiating it from thrombus or vegetation. ^8^^,^^9^

In the COVID-19 pandemic era, physicians should take extra precautions in both conducting diagnostic tests and managing PCTs. Multiple guidelines have been published on how to perform cardiac imaging tests safely in the COVID-19 pandemic era for the benefit of both physicians and patients. ^10^^,^^11^ Moreover, while surgery is usually the treatment of choice in myxomas, a simultaneous COVID-19 co-infection complicates matters as operating fundamentals and post-operative managements need to be readjusted. These variables need to be taken into account to improve the patient’s prognosis. We present a patient with a left atrial myxoma and concurrent delta variant COVID-19 infection, in accordance with the CARE reporting checklist.

## Case report

A 36-year-old Indonesian female patient was admitted to the emergency department with an initial complaint of atypical chest pain with an onset of 11 hours prior to admission. The patient felt a piercing pain on both hemithoraces without radiation to the jaw or left arm. Additional complaints included fatigue and shortness of breath, which worsened with activity and when the patient was lying in the supine position. The shortness of breath alleviated when the patient rested or when the patient was sitting. Since one month prior to hospital admission, the patient had been having trouble breathing, especially at night, and had to sleep with two to three pillows to alleviate the shortness of breath. The patient had also been losing weight, as she lost around 5 kg in the last 90 days prior to hospital admission. Around 90 days prior to hospital admission, the patient suddenly passed out for around 30 minutes while walking, preceded by light-headedness and headache. Upon waking up the patient lost the ability to move her left arm and leg. The next day the patient regained some of her motoric strength but had experienced hemiparesis and diplopia ever since. The patient had no comorbidities and upon further inquiry revealed a family history of cardiovascular disease.

**Figure 1.**
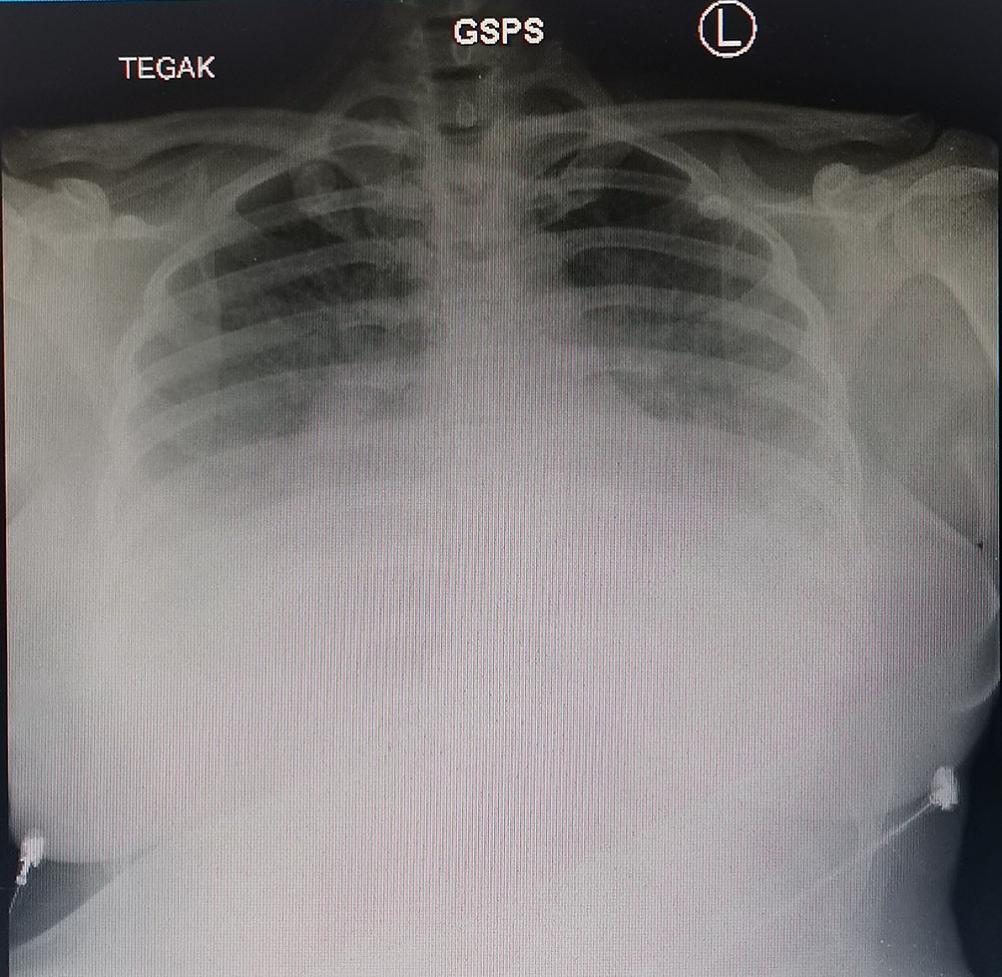
The patient's chest x-ray in the upright position.

**Figure 2.**
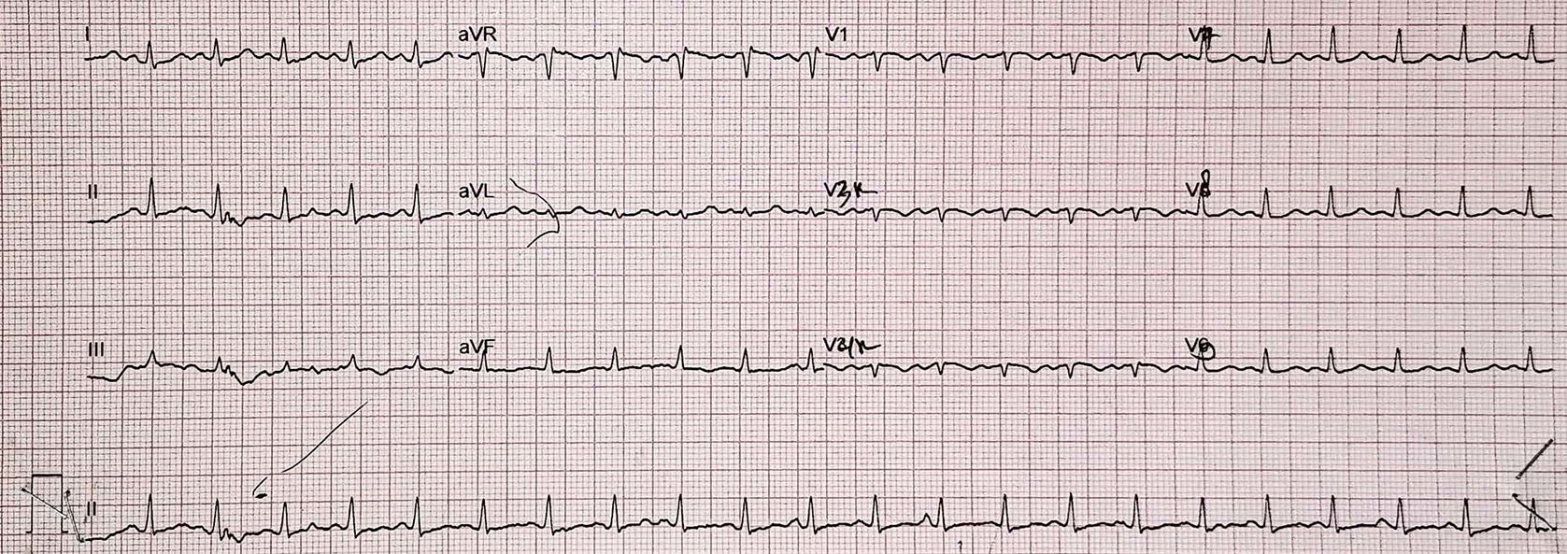
The patient's initial electrocardiogram in the ER.

Vital signs on admission showed a Glasgow Coma Scale (GCS) of E4M6V5, a body temperature of 36.8C, a respiratory rate of 30×/min, a heart rate of 118×/min, a blood pressure of 90/60 mmHg, and a peripheral oxygen saturation of 99% with supplemental oxygen of 3 lpm delivered through nasal cannula. Physical examination on admission showed presence of S4 gallop on cardiac auscultation, decreased breathing sounds on both bases of the lungs and generalized rhales more prominent on the left medial posterior lung on lung auscultation, and pitting oedema on both lower extremities. Laboratory examination on admission showed leucocytosis (14.9 × 10 ^9^/L) and thrombocytosis (680 × 10 ^9^/L), lowered serum calcium (7.4 mg/dL), increased fibrinogen levels (875 mg/dL), increased d-dimer levels (2,900 ng/mL), and increased C-reactive protein (CRP) levels (90.2 mg/L). Troponin T (<40 ng/L), Creatine Kinase (CK) (46 U/L), and Creatine Kinase-Myocardial Band (CK-MB) (15.6 U/L) was found to be normal. Blood gas analysis showed respiratory alkalosis, inadequately compensated (pH 7.518, pCO _2_ 23.8 mmHg, pO _2_ 111.50 mmHg, HCO _3_ 19.6 mmol/L, BE -1.30 mmol/L). Chest x-ray taken on the upright position showed bilateral massive pleural effusion with bilateral lung oedema ( Figure 1). Electrocardiography showed tachycardic sinus waves with a rate of 132 bpm, T wave inversion in lead III and V1, and ST segment depression in lead V5-V6 ( Figure 2). As part of the hospital’s admission policy, the patient was tested for COVID-19 using SARS-CoV-2 RT-PCR and the test came out negative.

**Figure 3.**
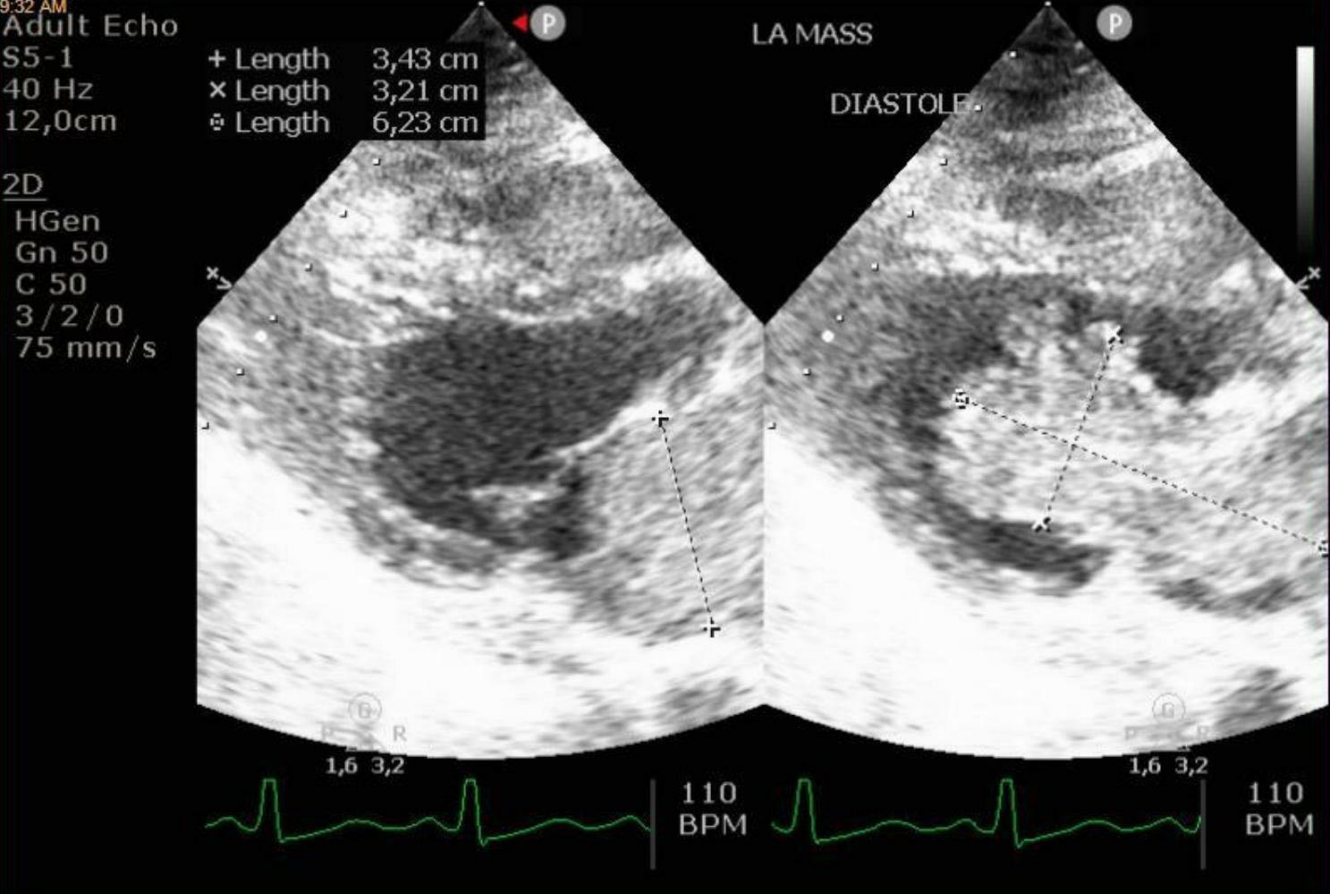
Two-dimensional transthoracic echocardiogram of the left atrial mass.

Working diagnoses established based on the acquired data were lateral non ST-elevated myocardial infarction (NSTEMI), warm & wet acute decompensated heart failure, bilateral pleural effusion, ischemic stroke, and hypocalcaemia. The patient received emergency care and was then immediately moved to the intensive cardiac care unit (ICCU) for further care and examination. In the ICCU, the patient received intravenous fluid drops (IVFD) of furosemide 5 mg/hr, subcutaneous (SC) enoxaparin sodium 0.6 mL bid, aspirin 80 mg qd, clopidogrel 75 mg qd, atorvastatin 40 mg qd, and intravenous (IV) calcium gluconate 1 g qd. On the second day of hospitalization troponin T was tested again and found to be elevated (125 ng/L). Pulmonary ultrasonography was also conducted and pleural puncture was subsequently done and a total of 50 mL of fluid was obtained. On day three of hospitalization the patient was given a control rate bisoprolol of 2.5 mg qd and transthoracic echocardiography was conducted. We found an oval-shaped, pedunculated mass with a dimension of 3.3 cm × 7 cm on the left atrium extending through the mitral valve into the left ventricle ( Figure 3).

The diagnosis of left atrial mass, highly suspected of myxoma, was established and a median sternotomy to evacuate and evaluate the mass was set to be scheduled as soon as a thorough neurological examination was done to assess overall procedure risk. On day seven of hospitalization, there was an alteration in the patient’s mental status as the patient started speaking incoherently and could sometimes be heard speaking to herself. The diagnosis of organic mental disorder was suspected and haloperidol 0.5 mg bid was added to the therapy regimen. The patient also experienced fever (>38C) and the patient was then tested for COVID-19 using SARS-CoV-2 RT-PCR as it was also a part of the hospital’s pre-operation policy for the scheduled procedure. On day eight of hospitalization the SARS-CoV-2 RT-PCR came out positive and the patient was confirmed for COVID-19. Confirmatory genomic analysis was subsequently done as part of the hospital epidemiologic surveillance. The delta variant of SARS CoV-2 was confirmed within three working days.

After the patient was confirmed for COVID-19, the pre-op assessment was halted and the patient was moved to the COVID-19 high care unit (HCU) to receive the standard therapy regimen for COVID-19. After twenty-two days, the COVID-19 infection resolved and the SARS-CoV-2 RT-PCR came out negative, the patient was re-evaluated for operation in order to evacuate the tumor, since the tumor had already shown signs and symptoms of obstruction. As soon as the septic marker and coagulation parameters were found to be within normal limits, the patient was scheduled for urgent operation. During anaesthetic induction, there was hemodynamic compromise, with elevated heart rate and supraventricular tachycardia (SVT). This unstable SVT occurred due to obstruction of the mitral valve. We were against cardioversion to prevent further embolization from the mass.

The sternotomy and bicaval was performed urgently. Cardiopulmonary bypass (CPB) was initiated via ascending aorta and bicaval cannulation, then cardiac arrest was obtained with cardioplegic solution. Topical cardiac hypothermia was applied for cardiac protection during operation. We used the right pulmonary vein as venting. The pericardium was preserved for interatrial patch. The approach to evacuate the mass was from the right atrium. An incision was made in fossa ovalis, then the pedicle of the tumor was seen along with the mass. The mass was irregular, homogenous, brown-greyish coloured with a dimension of 8×7×6 cm. It was also fragile, had a soft consistency and multiple pedicles. After further evaluation, we saw that the right upper pulmonary vein was obstructed by the mass. The mass was evacuated completely and the right atrium chamber was washed with normal saline to prevent microemboli from tumor remnants.

After evacuating the tumor, the mitral valve was evaluated with saline test and the result was remarkable, without any regurgitation or damage to the mitral valve leaflet. We did not perform mitral valve repair due to this finding. The interatrial septum was closed with pericardial patch due to the extensive mass extirpation. The CPB was weaned without any hemodynamic disturbances. Transoesophageal echocardiography was performed intraoperatively after the CPB was off, which showed trivial mitral regurgitation, mild aortic regurgitation, and no mitral valve regurgitation. Postoperative hemodynamic was unremarkable with low dose inotrope.

The mass was sent to the pathology department for histologic examination, and it was confirmed that the left atrial mass was indeed a myxoma. The patient was subsequently discharged 6 days after the procedure with outpatient medications, all taken orally: atorvastatin tablet 20 mg once daily, digoxin tablet 0.25 mg once daily, omeprazole capsule 20 mg once daily, warfarin tablet 2 mg once daily, furosemide tablet 20 mg twice daily, and bisoprolol tablet 2.5 mg once daily. The patient was able to communicate adequately and perform daily tasks (i.e eat and walk) with minimal assistance and without any neurological dysfunction post procedure.

## Discussion and conclusion

We present a middle-aged female with a left atrial mass highly suspected of myxoma and a subsequent concurrent delta variant COVID-19 infection confirmed a few days after hospitalization. To our knowledge, this is the first case report of a patient with a left atrial mass with a concurrent confirmed COVID-19 delta variant infection.

It is well documented that myxomas predominantly happen in females, more so in their productive years as we see in our patient. ^5^^,^^6^^,^^12^ The triad of clinical presentations of myxoma (haemodynamic consequences, systemic embolism, and constitutional manifestations) as described by Karabinis *et al*. ^6^ was also present in our patient in the form of dyspnoea, palpitations, congestive heart failure, fatigue, weight loss, and a history of cerebrovascular accident. In the case of cerebrovascular accidents, cardioembolic stroke in myxoma patients is a common phenomenon that usually happens in the middle cerebral artery. ^12^ Alvarez-Sabín *et al*. reported 11 of 28 (39.3%) cardiac myxoma patients had embolic phenomena, six of which affected the cerebral arteries (54.5%). ^13^ While our patient has regained some of her motoric strength, hemiparesis and diplopia are still present as of the writing of this paper. The neuroimaging exams’ results are still inconclusive. The patient’s progressively altered mental state could possibly be attributed to the evolution in size and/or location of the cerebral embolism. On the other hand, it is also probable that the systemic embolism in this patient is a case of COVID-19-associated thromboembolism, as COVID-19 patients are more predilected to develop thrombus due to their hypercoagulability. ^7^

Using TTE, we found an oval-shaped mass with a dimension of 3.3 cm × 7 cm in the left atrium extending through the mitral valve into the left ventricle. The diagnosis of thrombus and vegetation should always be excluded first in diagnosing myxoma. TTE has excellent sensitivity in detecting myxomas (95%), ^8^^,^^9^ while TEE has better sensitivity (100%) ^8^^,^^9^ than TTE and was considered, the idea was dropped due to COVID-19 restrictions as it is an aerosol-generating procedure. ^14^

Traditionally, simple tumor resection is the treatment of choice in patients with benign cardiac tumors such as myxoma, as the procedure has an excellent prognosis and is associated with low complications and recurrence rate. ^5^^,^^6^ However, patients with a concurrent COVID-19 infection such as ours complicate matters. A study by Sanders *et al*. found that patients with COVID-19 undergoing cardiac surgery had a significantly higher mortality rate (24.5% v 3.5%, p < 0.0001) and longer post-operative stay (11 days v 6 days, p = 0.001) than non-COVID patients. ^15^ Taking into account the patient’s COVID-19 infection and her stable condition, we decided to delay the procedure after the COVID-19 resolution.

Another interesting fact that we would like to point out is the positive outcome of the patient’s SARS-CoV-2 RT-PCR test eight days after hospital admission even though the patient was admitted with a negative SARS-CoV-2 RT-PCR result, tested again for the second time on the subsequent day of hospitalization, which also came out negative. A summary of the patient’s SARS-CoV-2 RT-PCR tests is available in Table 1. There are a few possibilities that we could think of that could have caused the patient to be infected with COVID-19 mid-hospitalization.

**Table 1.** Summary of the SARS-CoV-2 RT-PCR tests.

Timeline	Sample	Result
Day 1 of hospitalization	Naso-oropharyngeal swab	Negative
Day 2 of hospitalization	Naso-oropharyngeal swab	Negative
Day 8 of hospitalization	Naso-oropharyngeal swab	Positive

The first possibility is that the two initial SARS-CoV-2 RT-PCR results were false negatives. A study done by Gupta-Wright *et al.* studying all patients admitted to medical wards in two United Kingdom hospitals between 2 March and 3 May 2020 found that the real-life sensitivity of RT-PCR testing with nasopharyngeal specimens were only 83% when compared to the clinical reference in diagnosing COVID-19. ^16^ Challenges in hospital resources and logistics are suspected to be the main reasons. The second possibility is a nosocomial infection of COVID-19. While hospitals around the world have generally established protocols to avoid nosocomial COVID-19 infection, avoiding it completely has proven to be a challenge as reported by Du *et al*. ^17^ This case prompted the hospital to perform a thorough root cause analysis for its COVID-19 protocols to possibly improve the hospital’s prevention and control of infection.

In conclusion, although rare, patients admitted with haemodynamic compromise, systemic embolism, and constitutional manifestations should always be screened for cardiac tumors. TTE and TEE are excellent imaging modalities in detecting myxoma. Simple tumor resection is the treatment of choice for myxoma, but in the COVID-19 era, physicians should assess the risks and benefits more thoroughly while maintaining strict COVID protocols to get the best results while reducing the chance of nosocomial COVID-19 infection.

### Ethics

The authors are accountable for all aspects of the work in ensuring that questions related to the accuracy or integrity of any part of the work are appropriately investigated and resolved. All procedures performed in studies involving human participants were in accordance with the ethical standards of the institutional and/or national research committee(s) and with the Helsinki Declaration (as revised in 2013).

## Consent

Written informed consent was obtained from the patient for the publication of this case report and accompanying images.

## Data availability

### Underlying data

All data underlying the results are available as part of the article and no additional source data are required.

## Author contributions

SAN, EDT, MMA conceptualized and wrote the original draft of the article. EG, NDI, AP, MY, M, APS, MAP, CWP, IS, AFS, and LDL reviewed, wrote, and curated the data needed for the article.
