# Supplementary material for: Case Report: Left atrial myxoma with confirmed Delta variant COVID-19 infection, “to treat or withhold”
Source: F1000Res. 2022 Aug 31;11:986. [Version 1] doi: 10.12688/f1000research.124159.1 (PMC9490291; doi:10.12688/f1000research.124159.1)
Supplement: Peer review history

# Peer review: Reviewer response for version 1

**DOI:** 10.5256/f1000research.136337.r150285

**Published:** 2022-09-20

**Authors:** Kevin Wibawa

**Affiliations:** 1 Family Medicine, Gunung Jati General Hospital, Cirebon, Indonesia

This is an interesting case about left atrial myxoma and concomitant delta variant COVID-19 infection. The authors provided detail history taking, physical examination, patient's progression, and comprehensive treatment. Histopathologic result of the resected mass was also provided. Epidemiology data and the outcome of cardiac surgery complicated by COVID-19 infection supported and explained the approach that was used by the authors.

Are enough details provided of any physical examination and diagnostic tests, treatment given and outcomes?

Yes

Is the case presented with sufficient detail to be useful for other practitioners?

Yes

Is sufficient discussion included of the importance of the findings and their relevance to future understanding of disease processes, diagnosis or treatment?

Yes

Is the background of the case’s history and progression described in sufficient detail?

Yes

Reviewer Expertise:

Cardiovascular, critical care, and COVID-19

I confirm that I have read this submission and believe that I have an appropriate level of expertise to confirm that it is of an acceptable scientific standard.

# Peer review: Reviewer response for version 1

**DOI:** 10.5256/f1000research.136337.r148994

**Published:** 2022-09-01

**Authors:** George Samanidis

**Affiliations:** 1 First Department of Adult Cardiac Surgery, Onassis Cardiac Surgery Center, Athens, Greece

Thank you for your interesting report and your presentation. Some comments:

1. Please add an intraoperative photo of cardiac myxoma.
2. Please add some details about follow-up (1 or 2 or 3 months post-op)
3. Can you provide a preoperative chest CT scan? Please find the interesting information about pneumonia in patients with COVID in below references:
  Ye Z, Zhang Y, Wang Y, Huang Z, Song B. Chest CT manifestations of new coronavirus disease 2019 (COVID-19): a pictorial review. ^1^
  Tenda ED, Yulianti M, Asaf MM, Yunus RE, Septiyanti W, Wulani V, Pitoyo CW, Rumende CM, Setiati S. The Importance of Chest CT Scan in COVID-19. ^2^
4. How many days the patient stayed in the ICU?

Are enough details provided of any physical examination and diagnostic tests, treatment given and outcomes?

Partly

Is the case presented with sufficient detail to be useful for other practitioners?

Yes

Is sufficient discussion included of the importance of the findings and their relevance to future understanding of disease processes, diagnosis or treatment?

Yes

Is the background of the case’s history and progression described in sufficient detail?

Yes

Reviewer Expertise:

Cardiac Surgery

I confirm that I have read this submission and believe that I have an appropriate level of expertise to confirm that it is of an acceptable scientific standard.

## References

[1] : Chest CT manifestations of new coronavirus disease 2019 (COVID-19): a pictorial review. *Eur Radiol* .2020;30(8) : 10.1007/s00330-020-06801-0 4381-4389 10.1007/s00330-020-06801-0 32193638PMC7088323

[2] : The Importance of Chest CT Scan in COVID-19. *Acta Med Indones* .2020;52(1) :68-73 32291374
